# PDGF Receptor-α Does Not Promote HCMV Entry into Epithelial and Endothelial Cells but Increased Quantities Stimulate Entry by an Abnormal Pathway

**DOI:** 10.1371/journal.ppat.1002905

**Published:** 2012-09-13

**Authors:** Adam L. Vanarsdall, Todd W. Wisner, Hetian Lei, Andrius Kazlauskas, David C. Johnson

**Affiliations:** 1 Department of Molecular Microbiology and Immunology, Oregon Health and Science University, Portland, Oregon, United States of America; 2 The Schepens Eye Research Institute, Department of Ophthalmology, Harvard Medical School, Boston, Massachusetts, United States of America; University of Alabama at Birmingham, United States of America

## Abstract

Epidermal growth factor receptor (EGFR) and platelet-derived growth factor receptor-α (PDGFRα) were reported to mediate entry of HCMV, including HCMV lab strain AD169. AD169 cannot assemble gH/gL/UL128–131, a glycoprotein complex that is essential for HCMV entry into biologically important epithelial cells, endothelial cells, and monocyte-macrophages. Given this, it appeared incongruous that EGFR and PDGFRα play widespread roles in HCMV entry. Thus, we investigated whether PDGFRα and EGFR could promote entry of wild type HCMV strain TR. EGFR did not promote HCMV entry into any cell type. PDGFRα–transduction of epithelial and endothelial cells and several non-permissive cells markedly enhanced HCMV TR entry and surprisingly, promoted entry of HCMV mutants lacking gH/gL/UL128–131 into epithelial and endothelial cells. Entry of HCMV was not blocked by a panel of PDGFRα antibodies or the PDGFR ligand in fibroblasts, epithelial, or endothelial cells or by shRNA silencing of PDGFRα in epithelial cells. Moreover, HCMV glycoprotein induced cell-cell fusion was not increased when PDGFRα was expressed in cells. Together these results suggested that HCMV does not interact directly with PDGFRα. Instead, the enhanced entry produced by PDGFRα resulted from a novel entry pathway involving clathrin-independent, dynamin-dependent endocytosis of HCMV followed by low pH-independent fusion. When PDGFRα was expressed in cells, an HCMV lab strain escaped endosomes and tegument proteins reached the nucleus, but without PDGFRα virions were degraded. By contrast, wild type HCMV uses another pathway to enter epithelial cells involving macropinocytosis and low pH-dependent fusion, a pathway that lab strains (lacking gH/gL/UL128–131) cannot follow. Thus, PDGFRα does not act as a receptor for HCMV but increased PDGFRα alters cells, facilitating virus entry by an abnormal pathway. Given that PDGFRα increased infection of some cells to 90%, PDGFRα may be very useful in overcoming inefficient HCMV entry (even of lab strains) into the many difficult-to-infect cell types.

## Author Summary

Human cytomegalovirus (HCMV) causes substantial morbidity and mortality in immunocompromised patients and in developing infants. HCMV pathogenesis involves the capacity to infect many different cell types by multiple distinct entry pathways. Among the biologically important cell types infected *in vivo* are epithelial and endothelial cells. HCMV specifically requires the viral glycoprotein gH/gL/UL128–131 to enter these cells. Previous studies suggested that platelet derived growth factor receptor-α (PDGFRα) was important for HCMV entry into cells. We characterized whether PDGFRα was important for HCMV entry. Increased expression of PDGFRα in cells markedly augmented entry of wild type and gH/gL/UL128–131-mutant HCMV into epithelial and endothelial cells, however, other experiments showed that endogenous PDGFRα did not normally mediate HCMV entry into these cell types. Instead, the increased expression of PDGFRα increased HCMV entry by an abnormal pathway involving clathrin-independent endocytosis and low pH-independent fusion with endosomes. HCMV normally enters these cells by macropinocytosis and low pH-dependent fusion. Therefore, PDGFRα is not normally an HCMV entry mediator in these cells, but increased expression of PDGFRα can promote entry by a different pathway. PDGFRα transduction of cells may be very useful because many cells are poorly infected by HCMV and entry represents a major hurdle.

## Introduction

Human cytomegalovirus (HCMV) is a ubiquitous virus that establishes lifelong latency or persistence. HCMV normally causes only mild forms of disease, but in immunocompromised individuals HCMV can cause significant disease [1]–[3]. AIDS patients suffer from retinitis, a disease in which HCMV is left unchecked by reduced host cellular immunity and spreads between retinal epithelial cells and neurons destroying the retina. Immunosuppressed organ transplant patients suffer from HCMV infection of hepatocytes, lung and gut epithelial cells, and many other cell types. HCMV infection of placental trophoblasts leads to infection of the fetus and is associated with infection of microglial cells and defects in the developing nervous system [4]. Spread of HCMV in the blood involves infected monocyte-macrophages that can transmit virus to endothelial cells enabling viral spread into organs such as the gut, liver, lung, and brain [5]. Thus, *in vivo* HCMV infects a wide spectrum of cell types. However, in the laboratory, most studies are carried out with fibroblasts because the virus replicates relatively poorly in all other cultured cells.

Entry of HCMV into diverse cell types involves different entry pathways and viral entry mediators. HCMV binding to cell surfaces is initiated by interactions with highly charged heparan sulfate proteoglycans serving to concentrate virions on cell surfaces and promote other interactions with receptors that are more restricted in number [6], [7]. Entry into human fibroblasts involves direct fusion with the plasma membrane at neutral pH, delivering capsids into the cytoplasm [8]. By contrast, entry into epithelial and endothelial cells involves internalization of virions into endosomes and low pH-dependent fusion with endosomal membranes [9]. These two different pathways of entry require different complexes of HCMV gH/gL proteins.

HCMV assembles a glycoprotein complex composed of gH/gL decorated with three other proteins: UL128, UL130, and UL131 (gH/gL/UL128–131) that is required for entry into epithelial and endothelial cells, leuckocytes, and monocytes [9]–[15]. By contrast, gH/gL/UL128–131 is not required for entry into fibroblasts [9], [11], [16]. The assembly of the gH/gL/UL128–131 complex depends upon expression of all five proteins [12]. Extensive propagation of HCMV in fibroblasts produced mutations in the UL128, UL130, and UL131 genes so that laboratory strains, e.g. AD169, do not infect epithelial and endothelial cells and monocyte-macrophages because entry is blocked [9], [11], [17]. HCMV gH/gL also assembles with a third glycoprotein, gO, forming a disulfide linked trimer [18]. An HCMV TR gO-null mutant produced virus particles that had highly reduced quantities of gH/gL and were unable to enter fibroblasts and epithelial and endothelial cells [19]. Therefore, both gH/gL/UL128–131 and gH/gL/gO are important for entry of wild type or clinical HCMV into epithelial and endothelial cells, but only gH/gL/gO is necessary for entry into fibroblasts.

We used interference to support the hypothesis that gH/gL/gO and gH/gL/UL128–131 bind saturable receptors. Interference involves expressing viral receptor-binding glycoproteins in susceptible cells, so that cellular receptors are unavailable for virus entry [20]. When epithelial cells were transduced with gH/gL/UL128–131, the cells become resistant to HCMV entry [21]. Expression of gB, gH/gL or UL128–131 in epithelial cells did not produce interference. In fibroblasts, expression of gH/gL and gO produced interference, but expression of gH/gL/UL128–131 or gB did not interfere [21], [22]. These studies supported a working hypothesis suggesting that HCMV utilizes gH/gL/UL128–131 to interact with epithelial cell receptors, whereas gH/gL/gO serves to bind fibroblast receptors (reviewed in [23]). The requirement for HCMV gH/gL/UL128–131 correlates with entry involving low pH endosomes.

The HCMV glycoprotein gB is also essential for virus entry into fibroblasts and likely all cells [24]. Studies of the HSV and Epstein-Barr virus (EBV) gB homologues have produced evidence that gB is a fusion-inducing protein that is triggered by gH/gL molecules to mediate entry fusion [25], [26]. Moreover, HSV gB interacts directly with liposomes, while gH/gL does not [27], [28]. However, there have been reports that HCMV gB can act to bind cellular receptors. The epidermal growth factor receptor (EGFR) was described as an HCMV receptor and gB binds EGFR [29]. Activation of EGFR either by HCMV or gB binding produced cytoplasmic signals that apparently prepared cells for HCMV entry or early stages of virus replication. The role of EGFR in HCMV entry was challenged, leading to the conclusions that EGFR does not play a role in HCMV entry into fibroblasts, epithelial and endothelial cells and HCMV does not activate EGFR [30]. The platelet derived growth factor receptor-α (PDGFRα) was also reported as an HCMV receptor, promoting entry of both lab and clinical HCMV strains [31]. Again, there was evidence that HCMV, and specifically gB, caused phosphorylation of PDGFRα and downstream signaling via PDGFRα and this activation was important for entry or early events in virus replication. A PDGFRα–neutralizing monoclonal antibody (MAb) antibody, Gleevec (an inhibitor of PDGFRα kinase activity), and PDGFRα-specific siRNAs blocked HCMV infection of fibroblasts [31]. Integrins α2β1, α6β1, and αVβ3 have also been reported to promote HCMV entry into cells and gB binds integrins through a disintegrin domain [32]. This fits well with evidence that EBV and Kaposi's sarcoma herpesvirus utilize integrins in entry [33]–[35].

Reports that EGFR and PDGFRα can mediate entry of HCMV lab strain AD169 into fibroblasts, and in some cases into epithelial and endothelial cells, produced an apparent incongruity in the literature. AD169 lacks gH/gL/UL128–131 and cannot enter biologically important epithelial and endothelial cells and monocytes. Thus, it was not clear whether EGFR and PDGFRα were broadly important in entry of diverse cell types. We reasoned that there must be distinct pathways and other molecules that interact with gH/gL/UL128–131 to promote entry into epithelial and endothelial cells [21], [22]. We characterized whether clinical HCMV strain TR (that expresses gH/gL/UL128–131) relied on either EGFR or PDGFRα to enter epithelial and endothelial cells. We found no evidence that EGFR could promote entry of HCMV TR into any cell type. Transduction of PDGFRα into a variety of cell types including otherwise highly-resistant cells substantially increased TR entry. Surprisingly, increased PDGFRα also markedly promoted entry of HCMV lab strain AD169 and TR mutants unable to assemble gH/gL/UL128–131. A panel of PDGFRα-specific antibodies and PDGF (the ligand) did not inhibit HCMV entry into fibroblasts and epithelial and endothelial cells and silencing of PDGFRα did not reduce entry into epithelial cells, supporting the notion that PDGFRα is not normally required for virus entry into these cell types. Instead, increased expression of PDGFRα promoted a different pathway of endocytosis and pH-independent fusion, a pathway that does normally lead to HCMV entry into these cells.

## Results

### Expression of PDGFRα and EGFR using adenovirus (Ad) vectors

**Figure 1:**
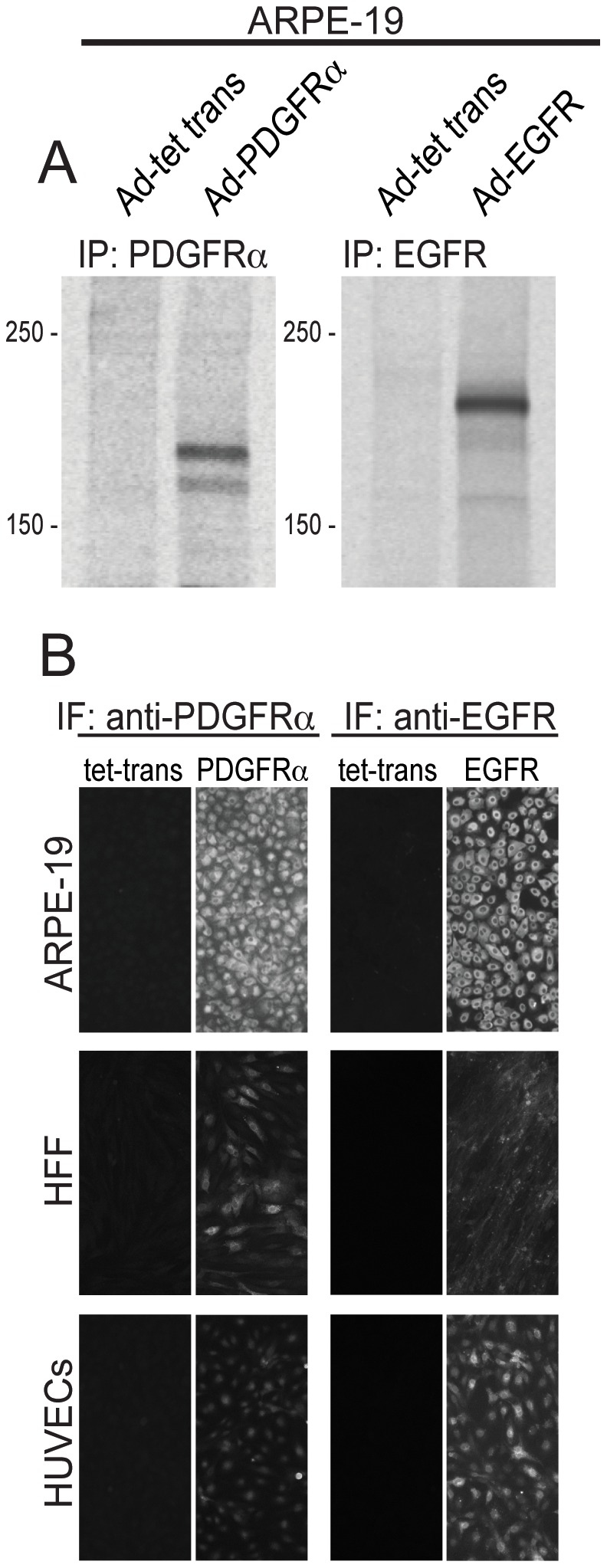
Characterization of Ad vectors expressing human PDGFRα and EGFR. (**A**) ARPE-19 epithelial cells were transduced with Ad-tet-trans alone (100 PFU/cell) or Ad-PDGFRα and Ad-tet-trans (50 PFU/cell of each) or Ad-EGFR and Ad-tet-trans (50 PFU/cell of each) for 24 hr then radiolabeled with [^35^S]-methionine-cysteine for 4 hr. The cells were lysed and the PDGFRα or EGFR proteins immunoprecipitated with a goat polyclonal antibody AF-307-NA or mouse MAb LA1, respectively. The precipitated proteins were analyzed by SDS-PAGE. Molecular weight markers are indicated on the left. IP refers to the protein immunoprecipitated. (**B**) ARPE-19 epithelial, human foreskin fibroblasts (HFF), and human endothelial cells (HUVECs) were transduced with Ad vectors expressing tet-trans, PDGFRα, or EGFR as described above, incubated for 24 hr then fixed and stained with the PDGFRα-specific antibody AF-307-NA or EGFR-specific antibody LA-1 followed by a FITC-conjugated donkey anti-goat (Molecular Probes) or a Dylight 594-congugated goat anti-mouse secondary antibody.

To investigate the roles of PDGFRα and EGFR in HCMV entry into a variety of cells, we constructed non-replicating (E1-) recombinant adenovirus (Ad) vectors. The PDGFRα or EGFR genes were coupled to a conditional promoter that was transactivated by co-transducing cells with a second Ad vector, Ad-tet-trans, that expresses a tetracycline transactivator protein as described [12], [36]. ARPE-19 retinal epithelial cells were transduced with non-replicating Ad vectors expressing PDGFRα or EGFR with Ad-tet-trans or with Ad-tet-trans alone for 24 hr and PDGFRα and EGFR were radiolabeled with [^35^S]-methionine-cysteine and immunoprecipitated. Both PDGFRα and EGFR were expressed well in these cells (Fig. 1A). It should be pointed out that ARPE-19 cells normally express PDGFRα [37] but this expression is significantly lower, compared with that attained following transduction with this Ad vector. To further characterize PDGFRα and EGFR expression, ARPE-19 epithelial cells, human foreskin fibroblasts (HFFs), and human umbilical cord vascular endothelial (HUVE) cells were transduced with Ad vectors for 24 hr then the cells fixed and stained with PDGFRα- or EGFR-specific antibodies in immunofluorescence experiments. For each cell type, there was significant expression of PDGFRα and EGFR in the vast majority of cells (Fig. 1B).

### Transduction of epithelial and endothelial cells with PDGFRα, but not EGFR, increases HCMV infection

**Figure 2:**
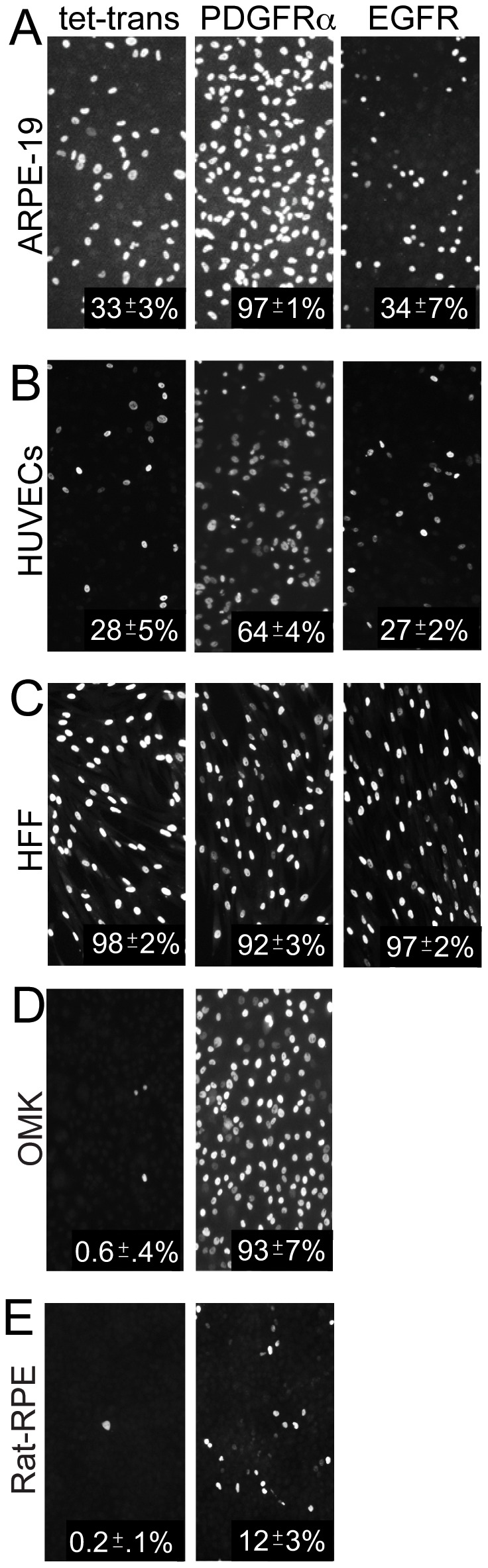
Expression of PDGFRα enhances entry of HCMV TR into epithelial and endothelial cells. **A**) Human epithelial cells (ARPE-19), **B**) human endothelial cells (HUVECs), **C**) human foreskin fibroblasts (HFF), **D**) owl monkey kidney cells (OMK), and **E**) rat retinal epithelial cells (Rat-RPE) were transduced with Ad vectors expressing tet-trans, PDGFRα, or EGFR using 50 PFU/cell for each Ad vector for 24 hr. The cells were infected with HCMV TR using 10 IU/cell and 1 IU/cell for HFF cells then fixed, permeabilized, and stained for HCMV IE-86 immediate early protein. The percent infected cells was calculated by counting the number of IE-86+ cells in three fields from three separate wells involving two separate experiments, comparing to the total number of cells in each field then averaging the numbers. Standard deviations are indicated.

To determine whether PDGFRα or EGFR would increase HCMV infection of ARPE-19 epithelial and HUVE cells, cells were transduced with Ad vectors to express PDGFRα or EGFR then incubated with HCMV clinical strain TR at 10 IU/cell. The percentage of cells that HCMV could enter was evaluated by assaying for the expression of the HCMV immediate early protein IE-86 by immunofluorescence. This measures productive entry, leading to infection of the cells, which is of utmost importance in these studies. ARPE-19 cells transduced with the control Ad vector (Ad-tet-trans) displayed 33% IE-86+ cells (Fig. 2A). ARPE-19 cells transduced to express EGFR were infected at similar levels. By contrast, ARPE-19 cells transduced with PDGFRα were much more extensively infected by HCMV, with 97% of the cells IE-86+ (Fig. 2A). Similar results were observed with HUVE cells, where 24% of the cells transduced with the control Ad-tet-trans vector were infected and there was no increase following transduction with EGFR (Fig. 2B). However, 64% of the HUVE cells transduced with PDGFRα were infected with HCMV (Fig. 2B). Human foreskin fibroblasts (HFF) cells were very efficiently infected with HCMV TR, so that cells transduced with the control Ad-tet-trans exhibited 98% IE-86+ cells using only 1 IU/cell, and this high level of infection was not changed when cells were transduced with PDGFRα or EGFR (Fig. 2C).

We also tested whether PDGFRα could enhance HCMV entry into non-permissive, non-human cells. HCMV infected only a small percentage of owl monkey kidney (OMK) cells and rat retinal pigmented epithelial (rat-RPE) cells transduced with the control Ad vector (Fig. 2D,E). That this defect in IE-86 expression involved defects in entry was previously demonstrated using polyethylene glycol (PEG), a chemical fusogenic agent that promoted IE-86 expression in the majority of cells [38]. Following transduction with PDGFRα, the numbers of IE-86+ OMK and rat-RPE cells was increased by 100 or 60-fold, respectively (Fig. 2D, E). We concluded that expression of PDGFRα can dramatically increase HCMV infection of human and non-human cells.

### PDFGRα enhances entry of HCMV unable to assemble gH/gL/UL128–131

**Figure 3:**
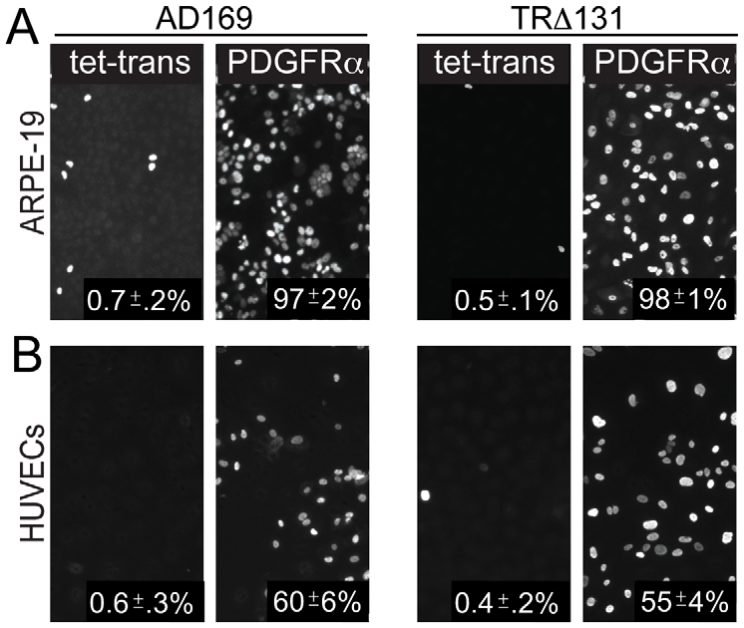
HCMV entry into PDGFRα expressing epithelial and endothelial does not require gH/gL/UL128–131 complexes. **A**) ARPE-19 epithelial cells or **B**) HUVECs were transduced with Ad-tet-trans or Ad-PDGFRα and Ad-tet-trans for 24 hr. The cells were then incubated with either HCMV lab strain AD169 or HCMV TR mutant virus TRΔ131 at 10 IU/cell for an additional 24 hr, then fixed, permeabilized, and assayed for HCMV infection by staining cells for the HCMV IE-86 early antigen. As in Fig. 2, the percent infected cells was calculated by counting IE-86 positive cells in three separate wells involving two experiments and the average number of IE-86+ cells indicated in the lower right of each panel.

We previously showed (using PEG) that HCMV mutants unable to assemble the five-protein complex gH/gL/UL128–131 are defective for entry into epithelial and endothelial cells [9]. As in the previous studies, AD169 and a TR mutant lacking the UL131 gene (TRΔ131), which can not assemble gH/gL/UL128–131, infected ARPE-19 cells transduced with the control Ad-tet-trans poorly, i.e. less than 1% of the cells expressed IE-86 (Fig. 3A). However, transduction of ARPE-19 cells with PDGFRα rendered the cells highly permissive for infection by both AD169 and TRΔ131, 97 and 98% of the cells were positive for IE-86, respectively (Fig. 3A). Similar effects were observed with TRΔ4 [9], a mutant lacking all of UL128, UL130 and UL131 (not shown). In addition, expression of PDGFRα in HUVE cells increased infection of AD169 and TRΔ131 by 100 and 137 fold, respectively (Fig. 3B). We concluded that increased expression of PDGFRα can greatly increase entry of both wild type HCMV and mutants lacking gH/gL/UL128–131 into epithelial and endothelial cells.

### Expression of PDGFRα overcomes gH/gL/UL128–131-mediated interference in epithelial cells

**Figure 4:**
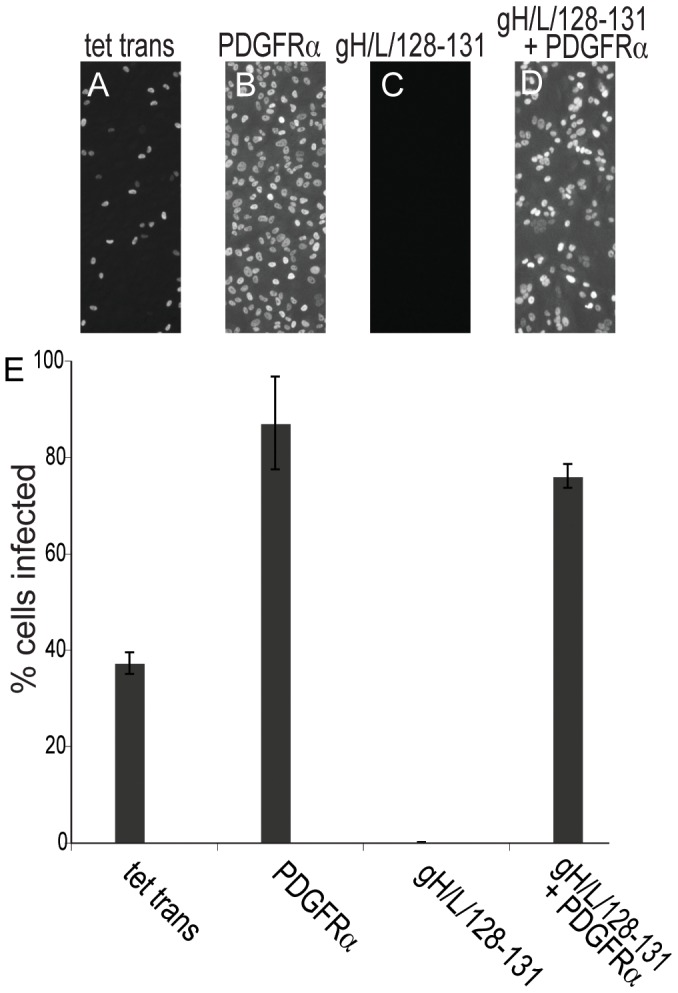
PDGFRα overcomes gH/gL/UL128–131 mediated interference in ARPE-19 epithelial cells. ARPE-19 epithelial cells were transduced with **A**) Ad-tet-trans alone, **B**) Ad-PDGFRα and Ad-tet-trans, **C**) Ad vectors expressing all of gH, gL, UL128, UL130 and UL131 and Ad-tet-trans, **D**) Ad- PDGFRα and vectors expressing gH, gL, UL128, UL130, UL131 and Ad-tet-trans for 24 hr. The cells were incubated with HCMV TR using 10 IU/cell for a further 24 hr, then the cells cells fixed, permeabilized, and stained for HCMV IE-86 early antigen. **E**) The percent of HCMV infected cells was quantified as described in Fig. 2. Error bars indicate the standard deviation.

Previously, expression of gH/gL/UL128–131 in ARPE-19 epithelial cells produced interference, consistent with our hypothesis that gH/gL/UL128–131 binds epithelial-specific receptors that are required for entry [21], [22]. Given observations that gH/gL/UL128–131 was not required for HCMV entry into ARPE-19 cells expressing PDGFRα, we tested whether gH/gL/UL128–131 would produce interference in ARPE-19 cells that also express PDGFRα. HCMV infected 37% of ARPE-19 cells transduced with the control Ad-tet-trans vector (Fig. 4A, E), whereas 90% of cells expressing PDGFRα were infected (Fig. 4B, E). There were no detectable HCMV infected cells expressing gH/gL/UL128–131, related to interference (Fig. 4C, E). However, ARPE-19 cells expressing both PDGFRα and gH/gL/UL128–131 were efficiently infected by HCMV, 78% of the cells expressed IE-86 (Fig. 4 D, E). Therefore, expression of PDGFRα in ARPE-19 overcomes gH/gL/UL128–131-mediated interference. Coupled with observations in the last section, this suggested that gH/gL/UL128–131 does not interact with PDGFRα and PDGFRα produces an alternate pathway of entry.

### The PDGF ligand and PDGFRα-specific antibodies do not reduce HCMV infection of epithelial, endothelial, and fibroblasts cells and silencing of PDGFRα in epithelial cells did not reduce entry

**Figure 5:**
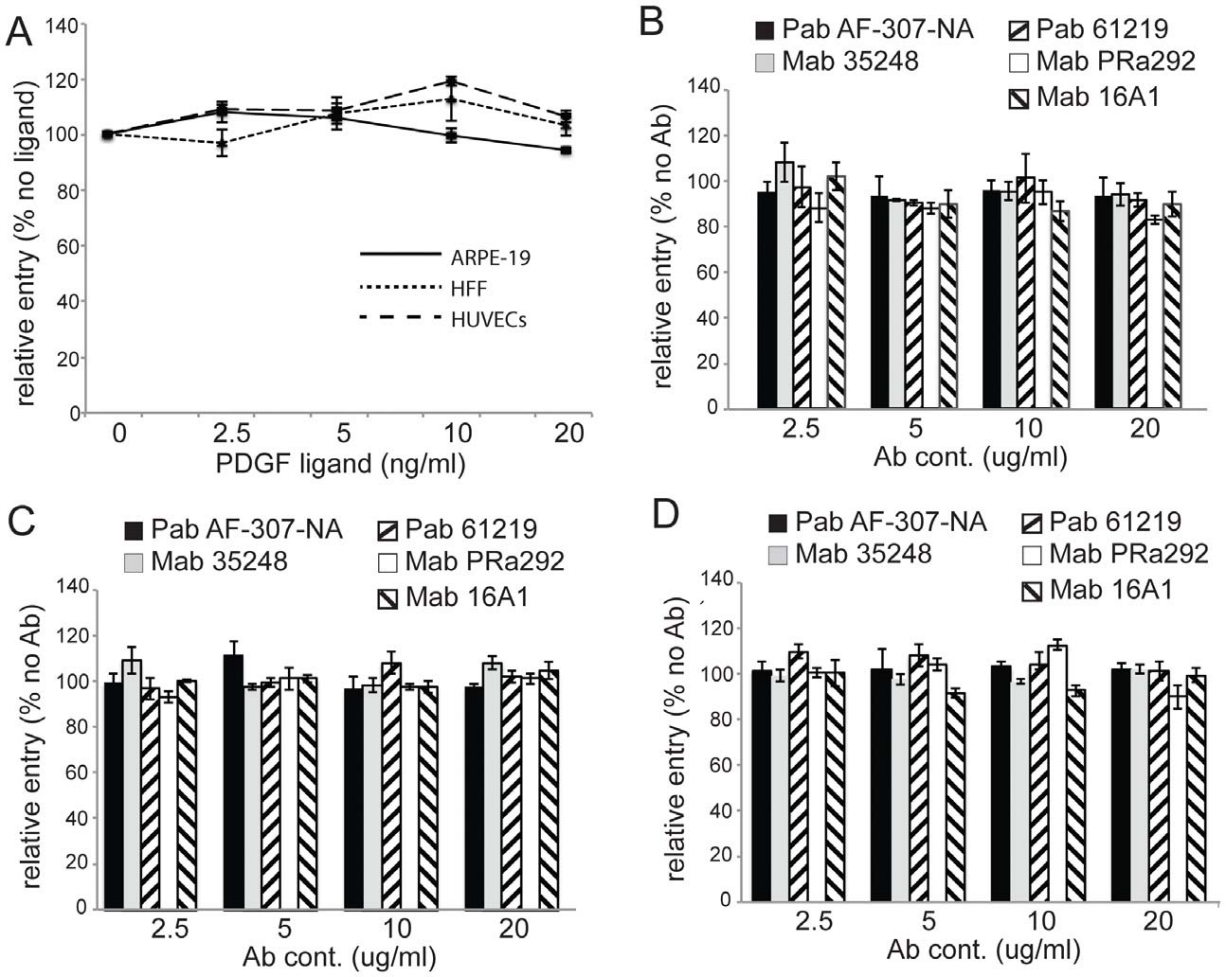
The PDGF ligand (PDGF-AA) and PDGFRα antibodies do not reduce HCMV infection of epithelial, endothelial cells, and fibroblasts. (**A**) ARPE-19, HUVECs, or foreskin fibroblasts (HFF) were pre-treated with PDGF-AA ligand at various concentrations at 4**°**C for 30 min then HCMV TR (10 IU/cell, 1 IU/cell for fibroblasts) was added to the cells in the presence of PDGF-AA at 4**°**C for an additional 30 min. The cells were shifted to 37**°**C for 4 hr then washed to remove the virus inoculum and fresh growth media containing the same concentration of PDGF-AA was added. After an additional 24 hr, the cells were fixed, permeabilized, and stained for HCMV IE-86. Relative entry was characterized by comparing the number of IE-86 positive cells in three separate fields with other dishes of cells not treated with PDGFR-AA. Error bars indicate the standard deviation. **B**) ARPE-19 cells, **C**) HUVE cells or **D**) human foreskin fibroblasts were incubated with various doses of PDGFRα-specific antibodies for 1 hr at 4°C then incubated with HCMV TR (10 IU/cell, 1 PFU/cell for fibroblasts) in the presence of these antibodies for an additional 1 hr at 4°C. The cells were then shifted to 37°C for 4 hr, washed to remove the virus inoculum then incubated at 37°C for 24 hr in the presence of antibodies. The cells were then stained for IE-86. Relative entry refers to the frequency of IE-86 positive cells compared to cells not treated with antibodies and was determined from at least three separate fields. Error bars indicate the standard deviation.

To further address whether PDGFRα plays a role in HCMV entry into normal cells, we attempted to block PDGFRα with antibodies and the PDGF ligand. Previously, it was reported that 10 ng/ml of the PDGFR ligand, PDGF-AA, inhibited HCMV infection of human embryonic lung fibroblasts [31]. Normal ARPE-19, HUVE cells, or HFFs were treated with various doses of PDGF-AA then infected with HCMV TR and assayed for HCMV IE-86 expression after 24 hr. There was no observed inhibition of HCMV entry into any of these cell types even at 20 ng/ml of PDGF-AA (Fig. 5A). To test whether PDGFRα-specific antibodies would block HCMV infection, ARPE-19 cells were pre-incubated with a panel of five different PDGFRα-specific antibodies then infected with HCMV TR and assayed for IE-86 expression after 24 hr. None of the PDGFRα-specific antibodies tested, including PDGFRα-neutralizing antibodies AF-307-NA and MAb 35248, reduced HCMV entry into ARPE-19 cells (Fig. 5B), HUVE cells (Fig. 5C), or fibroblasts (Fig. 5D).

**Figure 6:**
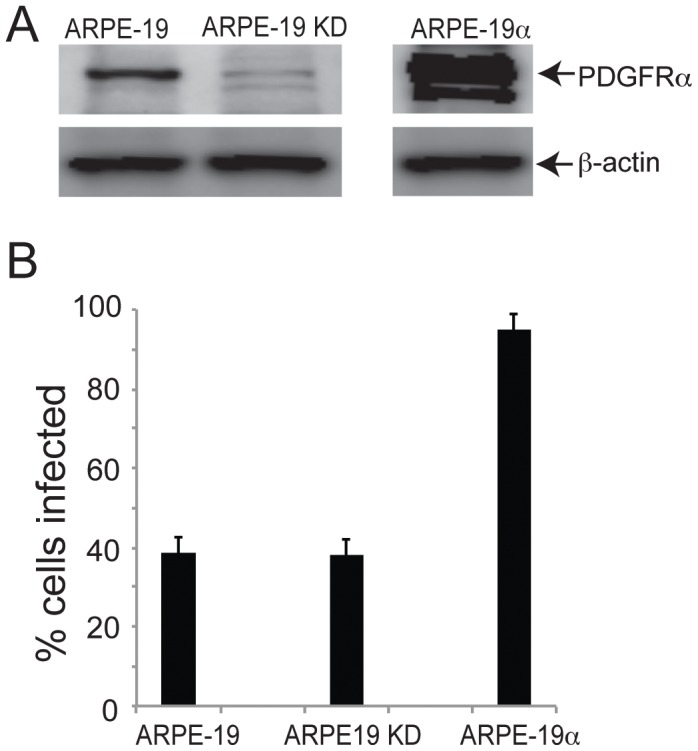
Reduction in expression of PDGFRα in ARPE-19 epithelial cells does not reduce HCMV infection. **A**) ARPE-19 cells, ARPE-19 cells transduced with a retrovirus expressing an shRNA targeting PDGFRα (ARPE-19 KD), or ARPE-19 cells transduced with a retrovirus causing overexpression of PDGFRα (ARPE-19α) were analyzed by staining western blots with a PDGFRα–specific polyclonal antibody. As a loading control, the same membrane was also probed for the cellular protein, β-actin with the mouse MAb AC-15. Note that the same gel was used to analyze PDGFRα in all three cell types, but a much stronger PDGFRα signal was expected for ARPE-19α cells and, thus, we separated the lanes in the figure. **B**) ARPE-19, ARPE-19 KD, and ARPE-19α cells were incubated with HCMV TR using 10 IU/cell for 24 hr then the cells were fixed, permeabilized, and stained for HCMV IE-86. The percent cells infected was derived by counting IE-86+ cells in at least three separate fields. Error bars represent the standard deviation.

To further investigate the role of PDGFRα in the normal route of entry of HCMV into epithelial cells, we characterized an ARPE-19 cell line, denoted ARPE-19 KD, which was transduced with a retrovirus vector expressing an shRNA to knockdown endogenous levels of PDGFRα [37]. Western blot analysis showed 80% reduction in PDGFRα expression in the ARPE-19 knockdown cells compared to normal ARPE-19 cells (Fig. 6A), as reported previously [37]. An ARPE-19 cell line that overexpresses PDGFRα following transduction with a retrovirus vector (ARPE-19α) was also characterized and shown to express 10–30-fold higher levels PDGFRα compared with normal APRE-19 cells (Fig. 6A). Note that we were unable to detect PDGFRα following radiolabeling of ARPE-19 cells in Fig. 1A. This difference relates to increased sensitivity of the Western blots. The ARPE-19α and ARPE-19 KD cells were tested for HCMV infection. Normal ARPE-19 cells displayed 39% IE-86+ cells 24 hr after HCMV infection and 38% of the ARPE-19 KD cells were infected. The ARPE-19α cells exhibited markedly increased numbers of IE-86+ cells (95%) as in previous experiments involving ARPE-19 cells transduced with Ad vectors. Together with the results of experiments involving PDGFRα-specific antibodies and the PDGFR ligand, these silencing experiments supported our conclusion that endogenous PDGFRα is not important for HCMV entry into cells.

### Gleevec inhibits HCMV IE-86 expression into ARPE-19 cells, but also blocks HSV IE protein expression

**Figure 7:**
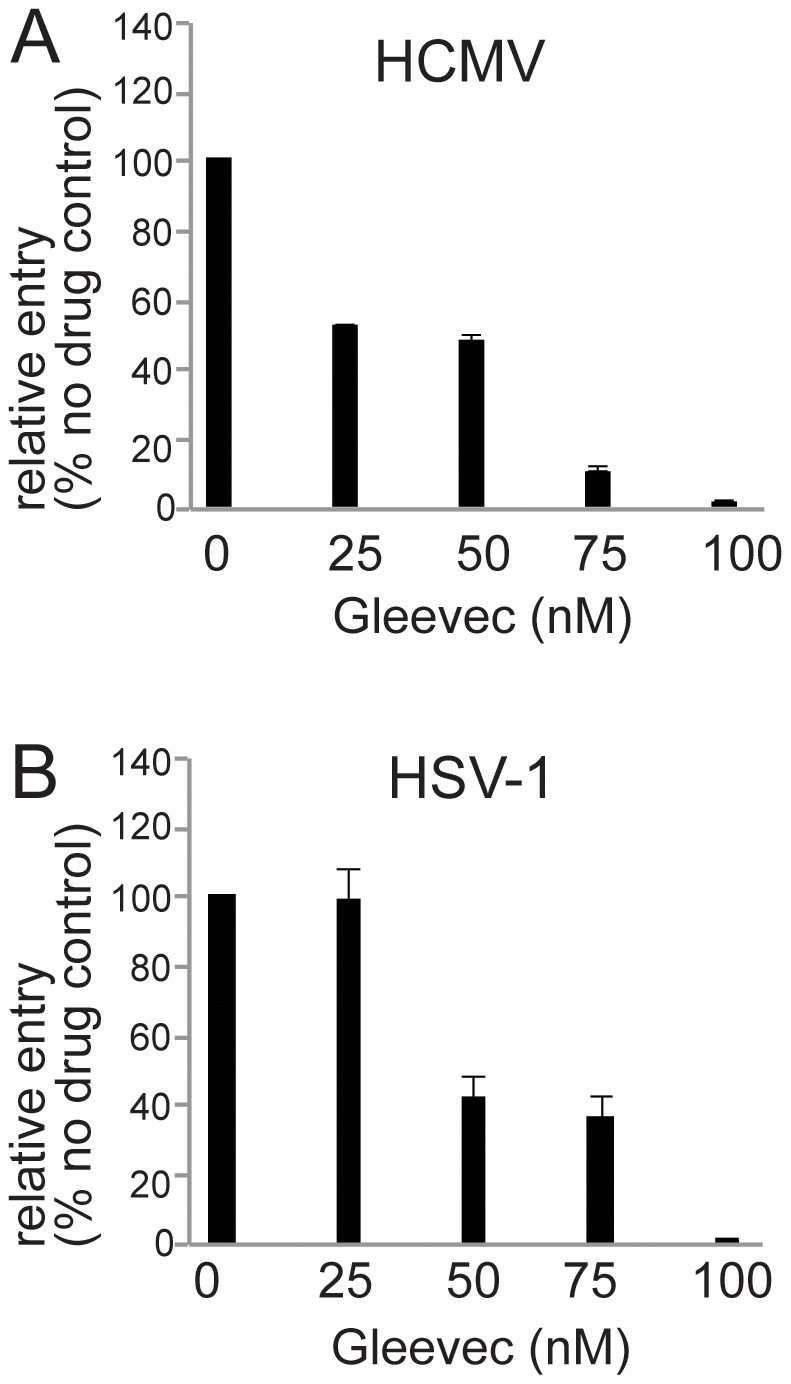
Gleevec inhibits both HCMV and HSV immediate early protein expression. Normal ARPE-19 cells were treated with various concentrations of Gleevec for 1 hr at 37**°**C then incubated with: **A**) HCMV TR using 10 IU/cell or **B**) HSV-1 using 10 PFU/cell in the presence of Gleevec for 4 hr at 37**°**C. The cells were washed to remove the virus inoculum then incubated for an additional 24 hr in growth media containing the same concentration of Gleevec. HCMV-infected cells were stained for IE-86 and HSV-infected cells were stained for immediate early protein ICP4. The percent infected cells was calculated by determining the percent IE-86+ or ICP4+ cells and comparing to the number of IE-86+ or ICP4+ cells observed when a different set of cells were treated with Gleevec after the virus inoculum was removed (at 4 hr post-infection). The numbers represent the average from at least three separate fields. Error bars represent the standard deviation.

In the previous report, the tyrosine kinase inhibitor imantinib methanesulfonate (Gleevec) substantially inhibited PDGFRα-mediated phosphorylation of Akt and HCMV IE expression in human embryonic lung (HEL) cells and U87 glioblastoma cells [31]. Thus, we pretreated cells with various doses of Gleevec prior to infecting the cells with HCMV TR in the presence of Gleevec. There was a 50–60% inhibition of the numbers HCMV IE-86+ cells when cells were treated with 25 and 50 nM Gleevec and 90–100% inhibition with 75 and 100 nM Gleevec (Fig. 7A). To determine whether this effect was specific for HCMV, Gleevec-treated ARPE-19 cells were infected with HSV-1. Gleevec at 50 and 75 nM concentrations produced ∼60% inhibition of the number of HSV immediate early ICP4 protein expressing cells and 100 nM Gleevec reduced HSV IE expressing cells to <1% (Fig. 7B). Because Gleevec inhibited both HSV and HCMV IE expression, Gleevec does not specifically affect HCMV entry and more likely has general effects on cells, likely reducing viral gene expression.

### PDGFRα expression does not increase HCMV glycoprotein-induced cell-cell fusion

**Figure 8:**
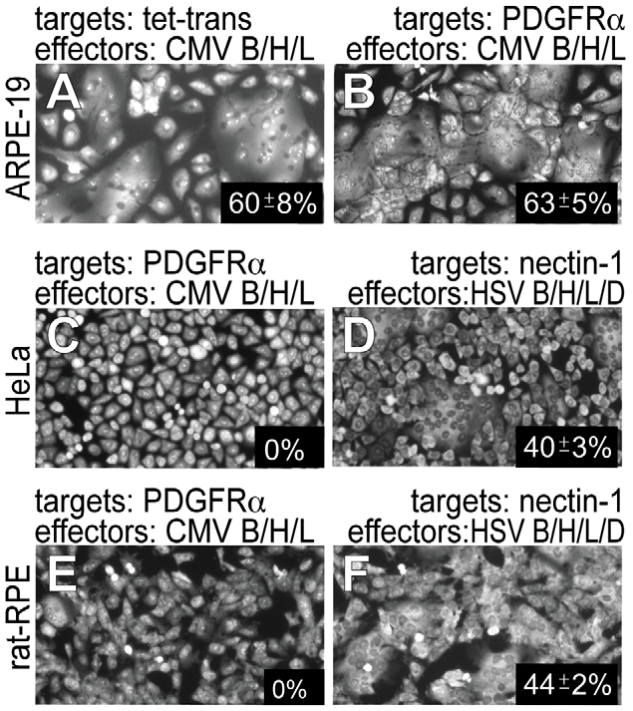
PDGFRα expression does not increase HCMV gB and gH/gL-induced cell-cell fusion. **A,** **B,** **C,** **and E**) Effector cells were established by transducing **A & B**) ARPE-19 cells, **C**) HeLa cells or **E**) rat-RPE cells with Ad vectors expressing HCMV gH, gL and gB and Ad-tet-trans. Target cells were established by transducing other cells of the same kind with **A**) Ad-tet-trans vector alone or **B, C, and E**) Ad-PDGFRα and Ad-tet-trans. **D**) HeLa effector cells were transduced with Ad vectors expressing HSV glycoproteins gB, gH, gL, and gD and HeLa target cells established by transducing cells with an Ad vector expressing nectin-1. **F**) rat-RPE effector cells were established by transduction with Ad vectors expressing HSV glycoproteins gB, gH, gL, and gD and rat-RPE target cells were established by transducing cells with an Ad vector expressing nectin-1. In every case, effector cells and target cells were trypsinized then replated in equal numbers in small dishes for 24–36 hr. To assess cell-cell fusion, cell monolayers were fixed and stained with syto-green dye (Invitrogen) and fusion was quantified from micrographic images by counting the number of nuclei involved in syncytia formation divided by the total number of nuclei. The percent of fused cells was calculated by counting fused cells versus unfused cells in 3 fields from 3 separate dishes from 2 experiments and involving over 500 cells and standard deviations indicated.

One important approach toward understanding virus entry has involved the delineation of viral glycoproteins and cellular proteins that promote cell-cell fusion. Studies with HSV showed that glycoproteins gB, gD, and gH/gL were all required both for cell-cell fusion and virus entry [39]–[45]. Importantly, cells lacking HSV receptors (HVEM and nectin-1) do not fuse and when transfected with HVEM or nectin-1 the cells become permissive for fusion [46]–[49]. We previously described HCMV TR gB and gH/gL as the minimal fusion machinery and observed a strong correlation between HCMV entry into human and non-human cells and whether the cells could fuse when HCMV gB and gH/gL were expressed in the cells [38]. As in the previous studies, a majority (60%) of ARPE-19 effector cells transduced with gB and gH/gL fused after mixing with ARPE-19 target cells transduced with the control Ad vector Ad-tet-trans (Fig. 8A). There was no significant increase in cell-cell fusion when ARPE-19 effector cells expressing gB and gH/gL were mixed with ARPE-19 target cells expressing PDGFRα (Fig. 8B). Previously, we reported that HeLa cells and rat-RPE cells did not fuse when the cells were transduced with HCMV gB and gH/gL and HCMV would not enter these cells [38]. To determine whether expression of PDGFRα promoted fusion of these cells, HeLa or rat-RPE effector cells expressing HCMV gB and gH/gL were mixed with HeLa or rat-RPE cells expressing PDGFRα. Under these conditions, no fusion was observed with either cell type (Fig. 8C, E). As a positive control and to establish a baseline level of glycoprotein-mediated fusion in these cells, HeLa and rat-RPE effector cells were transduced with Ad vectors expressing HSV-1 gB, gD and gH/gL then mixed with HeLa or rat-RPE target cells expressing the HSV-1 entry receptor nectin-1. Under these conditions, 40% of the HeLa cells fused (Fig. 8D), while 44% of rat RPE cells fused (Fig. 8F). We concluded that PDGFRα does not promote cell-cell fusion, supporting the conclusions that the HCMV fusion glycoproteins do not interact directly with PDGFRα and that PDGFRα does not act as a HCMV receptor.

### HCMV entry into ARPE-19 cells transduced with PDGFRα involves a different pathway compared with normal ARPE-19 cells

**Figure 9:**
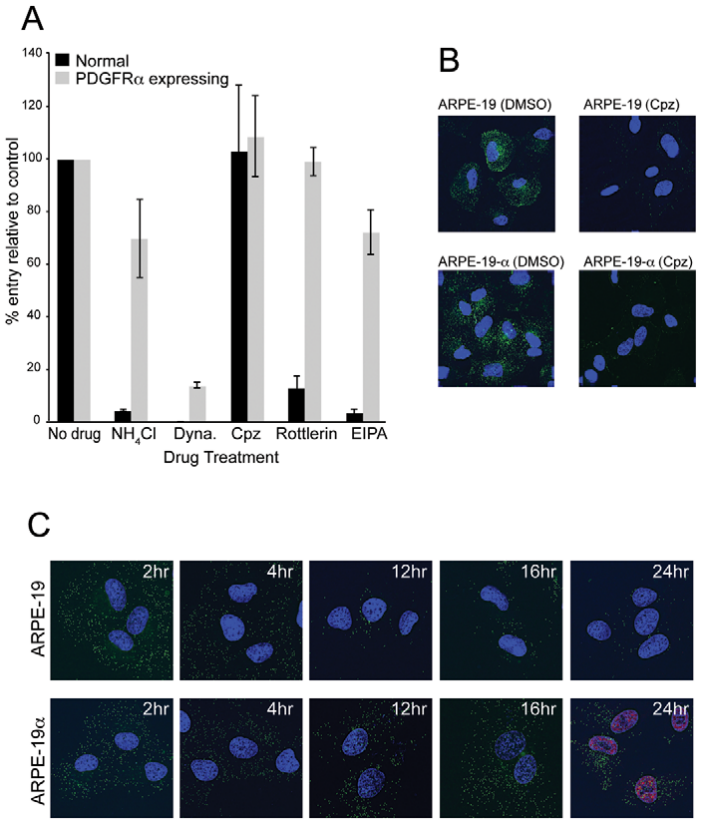
The pathway of HCMV entry into PDGFRα expressing epithelial cells is different from that involving normal ARPE-19 cells. **A**) ARPE-19 cells were transduced with Ad-tet-trans alone (Normal) or Ad-PDGFRα and Ad-tet-trans (PDGFRα expressing) for 24 hr at 37**°**C. The cells were incubated with 0.2% DMSO (No drug), 50 mM ammonium chloride (NH_4_Cl), 150 µM dynasore (Dyna), 30 µM chlorpromazine (Cpz), 30 µM Rottlerin, or 75 µM EIPA for 1 hr at 37**°**C then HCMV TR (10 IU/cell) was added for an additional 4 hr in the presence of drugs. The cells were then washed to remove the virus inoculum then incubated in media containing 10% FBS and the same concentration of drug for 24 hr at 37**°**C. The relative level of HCMV infection was measured by comparing the number of IE-86 positive cells in drug-treated cells with cells treated with 0.2% DMSO. The bars represent the averages from three separate wells from one experiment with standard deviations shown. Other experiments produced very similar data. **B**) ARPE-19 or ARPE-19α cells were treated with either 0.2% DMSO or with 30 µM chlorpromazine (Cpz) for 1 h at 37**°**C then incubated with media containing Alexa-fluor-488 transferrin and DMSO or chlorpromazine for 30 min at 37**°**C. The cells were placed on ice, washed briefly with citrate buffer, pH 3.0 to remove cell surface transferrin, fixed, the nuclei stained with DAPI, then analyzed by fluorescent microscopy. **C**) ARPE-19 or ARPE-19α cells seeded on glass coverslips were incubated with UL32-EGFP-HCMV using 10 IU/cell for 1 hr at 37°C then the cells washed and incubated for an additional 2, 4, 12, 16 hr in growth media before the cells were fixed, nuclei counterstained with DAPI, and cells analyzed by fluorescence microscopy. Cells incubated for 24 hr were fixed, permeabilized, and stained for the HCMV IE-86 early antigen along with an Alexa-fluor-594 secondary antibody and then counterstained with DAPI. Coverslips were mounted on glass slides and a 0.2 µm section of the Z-stack (including the central plane of the nucleus) was captured.

Previously, we reported that wild HCMV TR enters epithelial and endothelial cells by endocytic internalization and low pH-dependent fusion with endosomes [9]. Endocytosis was inferred from rapid internalization into epithelial cells and the requirement for low pH. Here, we characterized the pathways of entry into normal and PDGFRα-transduced ARPE-19 cells in more detail. Treating cells with NH_4_Cl (50 mM) raises endosomal pH, without causing cytotoxicity, and inhibited HCMV TR entry into normal ARPE-19 cells, but did not block entry into PDGFRα-transduced ARPE-19 cells (Fig. 9A). The small molecule inhibitor dynasore inhibits dynamin-2 that is crucial for endocytic vesicle formation in clathrin- and caveolin-mediated endocytosis [50], as well as more poorly understood clathrin- and caveolin-independent endocytic pathways [50], [51]. HCMV entry into both normal and PDGFRα-transduced ARPE-19 cells was reduced 88% or more by dynasore (Fig. 9A). Chlorpromazine which inhibits clathrin-mediated endocytosis [52], did not significantly inhibit HCMV entry into either normal or PDGFRα-transduced ARPE-19 cells. The amiloride analog, 5-(N-ethyl-N-isopropyl)-amiloride (EIPA) inhibits the Na+/H+ exchanger and specifically blocks macropinocytosis [53]. EIPA significantly inhibited HCMV entry into ARPE-19 cells, but had minimal effects on virus entry into ARPE-19 cells transduced with PDGFRα (Fig. 9A). Rottlerin, another inhibitor of macropinocytosis [54] had no effect on entry into PDGFRα-transduced ARPE-19 cells, but reduced entry into normal ARPE-19 cells.

Transferrin receptor (TfR) is normally taken up by clathrin-mediated endocytosis [55]. To measure whether PDGFRα transduction of cells increased TfR uptake, normal ARPE-19 or ARPE-19α (with increased PDGFRα) were incubated with fluorescent transferrin for 30 min at 37°C then cells briefly treated with pH 3.0 citrate buffer to remove cell surface transferrin and the cells imaged. There were no differences in the uptake of transferrin into cells in which PDGFRα was increased (Fig. 9B). When these cells were treated with chlorpromazine, fluorescent transferrin uptake into the cytoplasm was dramatically reduced (Fig. 9B). This experiment served as a positive control for the experiments in Fig. 9A, supporting the conclusion that HCMV does not enter normal or PDGFRα-transduced ARPE-19 cells by clathrin-mediated endocytosis.

We also measured whether PDGFRα increased the internalization of HCMV particles and delivery of a viral tegument protein to the nucleus. The HCMV recombinant, UL32-EGFP-HCMV, expresses a fluorescent tegument protein pp150-EGFP that produces fluorescent virons, as well as tegument puncta in the cytoplasm and nucleus following infection of fibroblasts [56]. It is important to note that UL32-EGFP-HCMV was derived from strain TB40, which was extensively passaged on fibroblasts and does not infect endothelial and epithelial cells well. Related to this, we showed that UL32-EGFP-HCMV was highly defective in producing IE-86 in ARPE-19 cells, compared with HCMV TR or TB40/E (not shown). Based on previous studies [57], it is highly likely that UL32-EGFP-HCMV cannot assemble gH/gL/UL128–131 explaining the inability to infect epithelial cells. UL32-EGFP-HCMV particles from cell culture supernatants were applied to normal or PDGFRα-transduced ARPE-19 cells for 1 hr at 37°C, then the cells washed and incubated for 2–24 hr at 37°C before imaging. After 2 and 4 hr, there were no obvious differences in the uptake of fluorescent particles into the cytoplasm of normal or PDGFRα-transduced cells (Fig. 9C). At 8 hr there was still no clear discernable differences in the number of particles (not shown). However, after 12, 16, and 24 hr, PDGFRα-transduced ARPE-19 cells exhibited substantial quantities of pp150-GFP puncta in the cytoplasm and in the nucleus. By contrast, there were few remaining pp150-GFP puncta in normal ARPE-19 cells after 12,16, and 24 hr and no fluorescent puncta in the nucleus (Fig. 9C.) We also looked at 24 hr post-infection to determine whether internalization of virus particles led to IE-86 expression. Whereas the internalization of particles in PDGFRα-transduced cells led to expression of IE-86, the very few pp150-GFP puncta that remained exclusively in the cytoplasm in normal ARPE-19 cells did not lead to expression of IE-86 (Fig. 9C). These results suggested that similar numbers of HCMV particles were taken up by endocytosis into both normal and PDGFRα-transduced cells. In PDGFRα-transduced cells, HCMV particles entered the cytoplasm and reached the nucleus, whereas in normal ARPE-19 cells, particles were degraded.

Together, the results in this section demonstrated that HCMV can be internalized into ARPE-19 cells by at least two endocytic pathways. Wild type HCMV particles containing gH/gL/UL128–131 can be internalized by macropinocytosis and can then fuse with endosomes requiring low pH. However, HCMV with or without gH/gL/UL128–131 can also be internalized by dynamin-dependent, clathrin-independent endocytosis. When cells are transduced to increase PDGFRα expression, this pathway leads to low pH-independent fusion and productive infection, whether virus particles possess gH/gL/UL128–131 or not. However, without increased PDGFRα expression, HCMV particles do not fuse with endosomes and are degraded, likely in lysosomes, disappearing from the cytoplasm as observed with UL32-EGFP-HCMV.

## Discussion

HCMV lab strains such as AD169 cannot assemble gH/gL/UL128–131 and cannot enter epithelial and endothelial cells. EGFR and PDGFRα were reported to mediate entry of HCMV AD169 into human fibroblasts, and for PDGFRα, entry into endothelial and epithelial cells. We reasoned that EGFR and PDGFRα cannot represent all of what is necessary for entry of wild type HCMV into epithelial and endothelial cells because this process requires gH/gL/UL128–131 and AD169 does not assemble this glycoprotein complex. Moreover, our interference data suggested that gH/gL/UL128–131 binds saturable receptors specifically expressed by epithelial cells and not by fibroblasts [21], [22]. EGFR and PDGFRα were increased in a variety of cells using Ad vectors. Ad transduction of EGFR did not increase entry of wild type HCMV TR into any cell type tested, fibroblasts, epithelial and endothelial cells. Moreover, EGFR-specific antibodies in the present studies (not shown) and our published studies did not inhibit HCMV cell-cell fusion and entry into any cell type [38]. Our conclusion that EGFR is not important for HCMV entry into fibroblasts, epithelial, and endothelial cells is similar to that in a previous report [30]. By contrast, Ad transduction of PDGFRα substantially enhanced entry of HCMV TR into human epithelial and endothelial cells, 97% of PDGFRα–transduced ARPE-19 cells were infected, compared with 33% of normal ARPE-19 cells. Expression of PDGFRα also substantially enhanced HCMV entry into owl monkey kidney cells and rat epithelial cells, cells that HCMV cannot normally infect. By contrast, entry of HSV-1 into rat epithelial cells was not enhanced by PDGFRα (not shown). These initial results suggested that PDGFRα was functioning as a bona fide entry receptor for HCMV in epithelial and endothelial cells.

However subsequent studies of the role of PDGFRα in HCMV entry quickly cast a different light on the question of whether this protein normally functions to promote HCMV entry into these cells. Surprisingly, expression of PDGFRα in epithelial and endothelial cells promoted high-level entry of HCMV mutants unable to assemble gH/gL/UL128–131, ie. a TR UL131- mutant entered 0.5% of normal ARPE-19 cells but 98% of PDGFRα-transduced ARPE-19 cells. This suggested that gH/gL/UL128–131 was not important for binding to PDGFRα or for HCMV to enter PDGFRα-transduced cells and contrasted with observations that gH/gL/UL128–131 is normally vital for entry into these cells [9]. Moreover, we observed no interference when gH/gL/UL128–131 was expressed in PDGFRα–expressing APRE-19 cells, in contrast to previous observations with normal ARPE-19 cells [21], [22]. Together, these observations argued that PDGFRα does not function in the normal pathway of HCMV entry into epithelial cells that requires gH/gL/UL128–131.

Additional evidence against PDGFRα acting as an important entry mediator in these cells came from studies involving antibodies and the PDGF ligand. Soroceanu et al. reported that the PDGF ligand and a PDGFRα–neutralizing MAb both inhibited HCMV entry into fibroblasts [31]. However, here, a panel of five different PDGFRα-specific antibodies, including two that neutralize PDGFRα, did not block HCMV entry into any of three cell types, fibroblasts, epithelial and endothelial cells, and the PDGF ligand had no effect on entry. Moreover, HCMV entered ARPE-19 cells transduced with PDGFRα–specific shRNA (reducing PDGFRα expression by ≈80%) to the same extent as with normal ARPE-19 cells. The antibody, PDGF ligand, and shRNA results all suggested that HCMV does not depend upon endogenous PDGFRα during entry into ARPE-19. Coupled with observations that gH/gL/UL128–131 is not necessary for entry into PDGFRα transduced cells, there must be other entry mediators that depend upon gH/gL/UL128–131. It was also reported that Gleevec, an inhibitor of PDGFRα–mediated signaling, inhibited HCMV entry into fibroblasts [31]. We found that Gleevec inhibited expression of both HCMV and HSV immediate early protein expression. Gleevec was developed as an inhibitor of the Abl tyrosine kinase and, thus, inhibits more than just PDGFRα. Therefore, it was not surprising that Gleevec caused more generalized effects in cells and the reduction of early gene expression was not specific to HCMV.

Further support for the notion that HCMV does not interact directly with PDGFRα came from cell-cell fusion studies. Increased PDGFRα did not increase cell-cell fusion involving ARPE-19 cells or HeLa and rat-RPE cells, cells that lack HCMV entry mediators and do not normally fuse [38], yet HeLa and rat-RPE cells fused with HSV glycoproteins. Previous studies of HSV and EBV cell-cell fusion demonstrated that virus receptors or entry mediators are absolutely required for cell-cell fusion [34], [46]–[49], [58]. Although unlikely, it is possible that PDGFRα interacts with other viral proteins, not gB or gH/gL, to promote fusion or entry.

**Figure 10:**
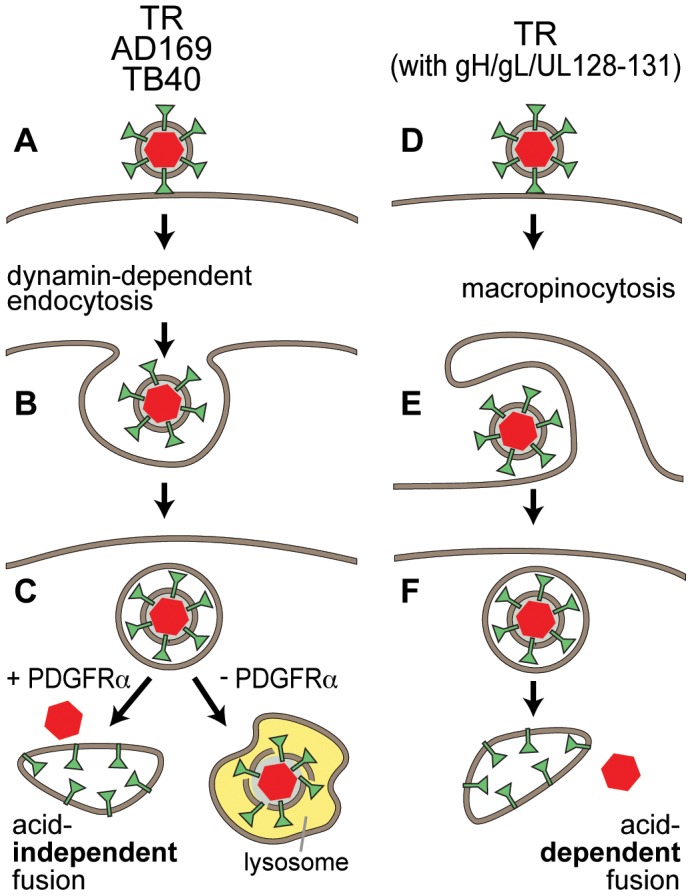
Models of HCMV entry pathways. **A–C). **HCMV TR, AD169 and TB40 are all taken up by dynamin-dependent endocytosis. If cells are induced to express increased PDGFRα, virus particles can fuse with endosomes by a low pH-independent mechanism, so that capsids enter the cytoplasm and can initiate infection after moving to nuclear pores. Without increased PDGFRα virus particles are degraded, presumably following delivery to lysosomes. **D–F**). Wild type HCMV TR particles which contain gH/gL/UL128–131 can also be taken up by macropinocytosis into endosomes. There is low pH-dependent fusion with endosomal membranes delivering capsids into the cytoplasm. It remains possible that AD169 and TB40 are also taken up by macropinocytosis but cannot exit endosomes derived from this process.

The pathway of entry of HCMV into PDGFRα transduced cells differed from that used by wild type HCMV to enter normal cells (depicted in Fig. 10). Wild type HCMV TR enters normal ARPE-19 cells by macropinocytosis (blocked by EIPA and rottlerin) and the low pH of endosomes (raised by NH_4_Cl) was required for entry fusion, i.e. access to the cytoplasm and induction of IE-86 (Fig. 10, right side). Dynasore specifically inhibits dynamin-mediated membrane scission and can block both endocytosis and macropinocytosis [59] and blocked entry of TR into normal ARPE-19 cells. Importantly, this pathway requires gH/gL/UL128–131, so that AD169 and TR mutants lacking UL128–131 cannot enter by this pathway. The left side of Fig. 10 depicts HCMV strains AD169 and TB40 (UL32-EGFP-HCMV), and likely TR, which can be internalized by another endocytic pathway requiring dynamin (inhibited by dynasore), but not requiring clathrin (not inhibited by chlorpromazine) and not involving macropinocytosis (not inhibited by EIPA and rottlerin) (Fig. 10, left). This pathway is apparently clathrin-independent endocytosis and does not require gH/gL/UL128–131, as evidenced by uptake of UL32-EGFP-HCMV and IE-86 expression by AD169. When PDGFRα expression is increased in cells, HCMV particles present in endosomes can fuse with endosomes, enter the cytoplasm, and allow viral DNA and tegument proteins to reach the nucleus. This pathway does not require low pH for fusion. Without increased expression of PDGFRα, UL32-EGFP-HCMV (TB40) and likely AD169 are unable to fuse with endosomes and degraded over time, apparently in lysosomes. This was supported by reduced numbers of pp150-EGFP fluorescent particles or puncta after 12 hr in normal ARPE-19 cells, compared with PDGFRα-expressing ARPE-19 cells and appearance of pp150-GFP in the nucleus.

It seems likely that endocytosis, macropinocytosis, and other types of internalization of virus particles occur simultaneously in most cells, but the majority of these processes do not result in productive entry, i.e. arrival in the cytoplasm, transport to nuclear pores and viral gene expression. For example, HSV particles that were applied to cells expressing gD (that interferes with entry) were internalized, remained in cytoplasmic vesicles, and were eventually degraded in lysosomes [60]. This was similar to the apparent fate of UL32-EGFP-HCMV particles in normal ARPE-19 cells. HCMV requires gH/gL/UL128–131 to access or exit from the endosomes engendered by macropinocytosis in normal ARPE-19 cells. However, virus particles lacking gH/gL/UL128–131 can access the alternate endocytic pathway when there is increased PDGFRα and these virus particles can fuse with endosomal membranes without low pH. It seems likely that increased PDGFRα signaling is responsible for these cytoplasmic steps. PDGFRα signaling involves dimerization, autophosphorylation, activation of tyrosine kinase activity, docking of other proteins and activation of a panel of partners including phospholipase C-γ, ras-MAP kinase, phosphatidylinositol 3-kinase (PI3K), and members of the src family of tyrosine kinases (reviewed in [61]). Important for our studies, PDGFRα activation affects both dynamin-2 that interacts with activated PDGFRα at the advancing edges of motile glioblastoma cells [62] and cortical actin cytoskeleton leading to marked membrane ruffling [63]. Thus, it is probable that PDGFRα increases HCMV entry through rearrangements of actin and, perhaps, dynamin-2 activity.

In summary, our studies have three important implications. First, we documented that both EGFR and PDGFRα are not important HCMV entry mediators in epithelial, endothelial, and fibroblast cells. These results help explain the incongruity in the literature involving the use of EGFR and PDGFRα by AD169 that lacks gH/gL/UL128–131. Coupled with our interference studies [21], [22], our results suggest other cellular molecules. However, importantly, it is very possible that HCMV enters other cell types, cells not studied here, involving these PDGFRα–mediated changes.

Second, our studies describe a very novel mechanism of action of PDGFRα that promotes entry of HCMV with or without gH/gL/UL128–131 by an abnormal pathway into both biologically relevant human epithelial and endothelial cells and several highly resistant cell types. We provided evidence that the HCMV entry machinery does not bind PDGFRα, instead PDGFRα alters endocytic machinery, allowing virus to escape endosomes that would otherwise be a dead end. There are extensive examples of herpesvirus entry into cells by different mechanisms, but none (that we know of) involving changes brought about in endosomal pathways related to expression of a signaling molecule that produces an entirely different pathway of entry. One could argue that the levels of PDGFRα that we expressed in these cells were not physiologically relevant. However, we observed huge increases in rates of virus entry (>90%) with a 30-fold increase in PDGFRα. Human cells can vary in the expression of nectin-1, an HSV receptor, by 10 fold [64].

Third, the results have potentially important implications for efforts to infect the numerous cell types that HCMV infects poorly, especially in the laboratory. HCMV entry into most cells, other than fibroblasts is poor, e.g. HCMV TR infects only 20–35% of epithelial and endothelial cells with 10 IU/cell (fibroblast infectious units), but this can be increased to up to 90% following PDGFRα transduction. Thus, PDGFRα transduction may markedly benefit studies of both HCMV lab and clinical strains in the numerous cell types that are difficult to infect.

## Materials and Methods

### Cells

Human neonatal foreskin fibroblasts (HFF) were obtained from Invitrogen, and grown in Dulbecco's modified Eagle's medium (DMEM) (Invitrogen) supplemented with 12% fetal bovine serum (FBS; HyClone). Human retinal pigmented epithelial (ARPE-19) cells were obtained from ATCC and grown in DMEM/F12 plus 10% FBS. Rat retinal pigmented epithelial cells (rat-RPE), owl monkey kidney (OMK), and HeLa cells were obtained from American Type Culture Collection (ATCC, Manassas, VA) and grown in Dulbecco's modified Eagle's medium (DMEM) (Invitrogen) supplemented with 10% fetal bovine serum. ARPE-19 cells that overexpressed human PDGFRα or ARPE-19 cells that were transduced with a retrovirus expressing an shRNA to PDGFRα were obtained from Hetian Lei at The Schepens Eye Research Institute, Harvard Medical School, Boston, MA. Human umbilical cord vascular endothelial cells (HUVECs) were a kind gift from Ashley Moses at the Vaccine and Gene Therapy Institute in Portland, Oregon and were maintained in Medium-200 plus low serum growth supplement (LSGS) (Invitrogen). 293 M cells were obtained from Microbix Biosystems (Toronto, Ontario) and were grown in minimal essential medium (MEM, Invitrogen) plus 10% FBS. Human retinoblastoma cells (911) were provided by RC Hoeben at the University of Leiden, Netherlands, and grown in (DMEM) supplemented with 10% fetal bovine serum. All cells were maintained at 37°C in 5% CO_2_ with the exception of rat-RPE cells which were maintained at 33°C in 5% CO_2_.

### Adenovirus vectors

Non-replicating adenovirus vectors expressing PDGFRα and EGFR, were constructed from plasmid Ad-X-ccdB that was modified to allow compatibility with the Gateway cloning technology (Invitrogen). Ad-X-ccdB harbors the adenovirus 5 genome and has a ccdB suicide gene flanked by attP1 and attP2 sites just down stream of the tetracycline induced promoter located in the E1 coding region. Genes containing compatible attB1 and attB2 sites can be efficiently recombined into this locus using BP clonase (Invitrogen). To construct Ad-X-ccdB, a DNA fragment containing attP1 and attP2 sites and the ccdB gene was isolated from plasmid pDONR222 (Invitrogen) with the restriction enzymes HpaI and HindIII and then ligated into the SmaI and HindIII sites of the shuttle vector pDNR-CMV (Clontech). This pDNR-CMV shuttle plasmid was then used with the parental adenovirus plasmid Adeno-X, (Clontech) in a cre-lox reaction according to the manufacturer's instructions. The plasmid DNA was transformed into one-shot ccdB survival cells (Invitrogen) and bacterial colonies were selected on LB agar plates containing chloramphenicol and ampicillin. Individual colonies were screened for the presence of the recombinant adenovirus plasmid containing the attP sites and the ccdB by extracting and analyzing plasmid DNA by restriction digestion with BsrGI. A positive clone was selected and used to construct PDGFRα and EGFR expressing vectors. To accomplish this, human cDNAs encoding PDGFRα (Accession # BC0634414) or EGFR (Accession # BC094761) in plasmid pCMV-SPORT6 (Invitrogen) were purchased from Open Biosystems. These plasmids were then used for BP-mediated recombination (Invitrogen) with the Adeno-X-ccdB plasmid according to the manufacture's instructions. After *in vitro* recombination, the DNA was electroporated into DH10B electrocompentent cells and recombinant constructs were selected on LB agar plates containing chloramphenicol and ampicillin. Individual colonies were screened for the presence of recombinant adenoviruses by extracting and analyzing plasmid DNA by restriction digestion with BsrGI. This method was also used to generate an adenovirus vector expressing human nectin-1 after the nectin-1 gene was excised from plasmid pBG38 and sub-cloned into the pCMV-SPORT6 plasmid. The resulting recombinants termed Ad-X- PDGFRα, Ad-X-EGFR, and Ad-X-Nectin-1 were then used to generate recombinant viruses. For this, Ad-X-PDFRA, Ad-X-EGFR DNA, and Ad-X-Nectin-1 DNA was purified from bacteria cultures using Qiagen columns, digested with PacI (New England Biolabs) to linearize the adenovirus DNA, and then transfected into 911 retinoblastoma cells by calcium phosphate transfection. Cells were monitored for 4–6 days for the presence of adenovirus induced CPE, at which point total cell lysates containing infectious adenovirus were generated by harvesting cells and lysing by sonication. Cell lysates were stored at −80°C until future use. Adenovirus vectors expressing the tetracycline transactivator (Ad-tet-trans), HCMV gB, gH, gL, UL128, UL130, UL131, and HSV-1 gB, gH, gL, and gD were constructed as described previously [12], [38] All large scale adenovirus vector stocks were generated by infecting 293 M cells (Microbix) at 0.1 plaque forming unit (PFU)/cell. Cells were harvested 6–10 days after infection and centrifuged at 800× g for 5 min. Cell pellets were suspended in DMEM plus 10% FBS, sonicated to release cell-associated virus, followed by centrifugation at 3000× g for 5 min to remove large cellular debris. Virus-containing cell lysates were stored at −80°C. Ad stock titers were determined by plaque assays on 293 M cells.

### HCMV

HCMV TR is a clinical HCMV strain that was derived from an ocular vitreous fluid sample from a patient with HIV disease and was cloned into a BAC after limited passage in fibroblasts [65], [66]. The HCMV strain AD169 was provided by Jay Nelson at the Vaccine and Gene Therapy Institute in Portland, Oregon. Construction of TR Δ4 and TR Δ131 has been described previously [9]. HCMV VR-1578 derived from TB40/E and expressing a fluorescent tegument protein (pp150-EGFP) [56] was obtained from the American Type Culture Collection. HCMV stocks were produced from HFF cells grown in roller bottles and viral particles were purified and concentrated from culture supernatants by centrifugation through a cushion of 20% sorbitol in PBS at 50,000×g for 1 h. Pellets were resuspended in DMEM plus 10% FBS and frozen at −70°C. Stocks were titered by determining the number of infectious units per ml (IU/ml) by plating serial dilutions of virus preparations on HFF cells and staining for IE-86 after 24 hr as described below.

### Reagents and antibodies

The chemical inhibitors Dynasore hydrate, Chlorpromazine hydrochloride, Rottlerin, and 5-(N-Ethyl-N-isopropyl) amiloride (EIPA) were obtained from Sigma-Aldrich. Imantinib Methanesulfonate (Gleevec) was obtained from LC laboratories (Woodburn, MA). The MAb clone LA1 (neutralizing) to EGFR and the rabbit anti-phospho-PDGFRα (Tyr-742) antibody was obtained from Millipore (Temecula, CA). The rabbit polyclonal antibody 61219 to PDGFRα was obtained from Abcam (Cambridge, MA). Human recombinant platelet-derived growth factor (PDGF-AA) and the mouse MAbs 35248 (neutralizing) and PRa292 as well as the polyclonal antibody AF-307-NA (neutralizing) to PDGFRα was obtained from R & D Systems (Minneapolis, MN). The MAb 16A1 to PDGFRα was obtained from NOVUS biologicals (Littleton, CO). The polyclonal antibody 31614 used in western blots to detect PDGFRα was obtained from cell signaling technology, (Danvers, MA). The rabbit polyclonal antibody R638 to HCMV IE-86 [67] was a kind gift of Jay Nelson (O.H.S.U.) and the mouse MAb 58S to HSV-1 ICP4 [68] was a gift of Roger Everett (University of Glasgow).

### Immunofluorescence staining

To stain for specific antigens, cells were fixed with PBS containing 2% formaldehyde for 10 min at room temperature then permeabilized with immunofluorescence (IF) buffer (PBS supplemented with 0.5% Triton X-100, 0.5% deoxycholate, 2% goat serum, and 0.05% sodium azide) for 30 min before incubation with primary antibodies diluted in IF buffer for 1 h. To detect PDGFRα and EGFR expression, cells were stained with the polyclonal AF-307-NA or MAb LA1, respectively. To detect HCMV or HSV-1 infected cells, cell monolayers were stained with the primary antibodies R638 or 58S that recognize the HCMV IE-86 and HSV-1 ICP4, respectively. After primary antibody incubation, cells were then washed several times with IF buffer and stained with a Dylight 594-goat anti-mouse, Dylight 594-goat anti-rabbit, or FITC-conjugated donkey anti-goat secondary antibodies in IF buffer for 1 h. The nuclei were then counterstained by incubating the cells in PBS containing DAPI (4′,6-diamidino-2-phenylindole) for 20 min and then the glass coverslips were mounted on glass slides with fluoromount-G (SouthernBiotech). Immunofluorescence microscopy was performed on a Nikon TE 200-based Applied Precision Instruments (API) Deltavision image restoration system.

### HCMV entry assays

Cell monolayers seeded in 24-well culture dishes were transduced with Ad vectors for 24 hr then infected with purified HCMV using 10 IU/cell (1 IU/cell for fibroblasts) in DMEM containing 2% FBS at 37**°**C for 4 h. After 4 hr, the cells were washed once with PBS to remove the virus inoculum and then incubated in growth media (DMEM containing 10% FBS) for 24 hr. For PDGF-AA ligand competition assays, cells were pre-incubated in infection media containing PDGF ligand at 4**°**C for 30 min then HCMV virions added at 10 IU/cell for an additional 30 min at 4**°**C. The cells were then incubated at 37**°**C for 4 hr, the virus inoculum was removed, the cells washed once with PBS, and incubated in fresh growth media containing the same concentration of ligand at 37**°**C with for 24 hr. For antibody inhibition assays, cells in media containing 2% FBS and various concentrations of PDGFRα-specific antibodies were incubated at 4**°**C for 1 hr then HCMV virions at 10 IU/cell added for 4 hr at 37**°**C. The cells were then washed once with PBS and incubated in fresh media containing 10% FBS and the same concentration of PDGFRα-specific antibodies at 37**°**C for 24 hr. For infection assays performed in the presence of drugs or chemical inhibitors, cells were pre-incubated in media containing 2% FBS and chemical inhibitors or drugs for 1 hr at 37**°**C then infected with purified HCMV virions using 10 IU/cell at 37**°**C for 4 h in the presence of drugs. The virus inoculum was removed, the cells washed once with PBS, then fresh media containing 10% FBS and the same concentration of drugs or inhibitors. To monitor for drug or chemical induced cytotoxicity (postinfection), cells were infected with HCMV for 4 hr to allow virus entry then drugs or chemicals were then added for 24 h. At the end of these 24 hr infections, HCMV IE-86 was measured by immunofluorescence as described above.

### Internalization and nuclear transport of UL32-EGFP-HCMV

Entry assays involving UL32-EGFP-HCMV were performed using ARPE-19 cells seeded on glass coverslips. Cells were incubated with UL32-EGFP-HCMV supernatant stock using 10 IU/cell for 1 hr. The cells were washed once with PBS and allowed to continue to incubate in fresh media containing 10% FBS for 4,6, 8, and 12 h then the cells were fixed with PBS containing 4% paraformaldehyde, counterstained with DAPI, mounted on glass slides, and analyzed as by immunofluorescence microscopy as described above.

### Transferrin uptake

ARPE-19 cells seeded on glass coverslips were treated with 30 uM chlorpromazine in serum-free media at 37**°**C for 1 hr. The media was then replaced with fresh serum-free media containing 0.1 mg/ml of Alexa-fluor-488 conjugated transferrin (Invitrogen) and the cells were incubated at 37**°**C for 30 min. The cells were then placed on ice, washed once with citrate buffer (40 mM citric acid, 10 mM KCl, 135 mM NaCl, pH 3.0) and then fixed with PBS containing 4% paraformaldehyde and nuclei counterstained with DAPI then cells characterized by immunofluorescence microscopy as described above.

### Interference assays

ARPE-19 epithelial cells seeded in 24-well culture dishes were transduced with non-replicating Ad vectors expressing tet-trans, the HCMV glycoproteins gH/gL/UL128-131 or PDGFRα. Tet-trans and the HCMV glycoprotein expressing Ad vectors and the Ad-PDGFRα vector were used at 50 PFU/cell. For conditions in which cells expressed tet-trans, PDGFRα or the gH/L/UL128-131 alone, an Ad vector expressing GFP (Ad-GFP) was added at different amounts so that the total number of PFUs for ARPE-19 cells was 350. The cells were allowed to incubate for 24 h to allow for adequate protein expression and then infected with HCMV TR at 10 IU/cell. At 24 h after infection with HCMV, entry was analyzed by immunofluorescence staining for HCMV IE-86 as described above.

### Cell-cell fusion assays

Target and effector cells were generated by transducing cells with Ad vectors at 50 PFU/cell. At 16 h post-transduction, the cells were washed thoroughly with PBS and trypsinized with 0.025% trypsin/EDTA buffer. After the cells were released from the dish, the cells were counted and an equal number of target and effector cells were mixed and re-plated onto culture dishes and maintained in growth media for an additional 24-36 h. To assess the level of fusion, cells were fixed in PBS containing 2% formaldehyde, permeabilized with PBS containing 0.2% triton X-100, and stained with a 5 um stock of SYTO 13 green fluorescent nucleic acid stain (Invitrogen) diluted 1∶20,000 in PBS. Images were captured on Nikon eclipse TS100 microscope fitted with a Qiacam digital CCD camera and the level of fusion was quantified by counting the total number of cell nuclei involved in syncytia formation divided by the total number of nuclei in the same field and expressed as the percentage of cells fused.

### Radiolabeling cells and immunoprecipitation

Cells were labeled with with [^35^S]-methionine/cysteine by first washing in medium lacking methionine and cysteine followed by incubation in this media for 30 min at 37° C. The cells were then incubated in labeling medium supplemented with [^35^S]-methionine/cysteine (300 µCi/ml; Amersham) for 4 h at 37° C. Extracts of labeled cells were made using 1% NP40 in PBS supplemented with 1 mg/ml bovine serum albumin and 1 mM phenylmethylsulfonyl fluoride. Extracts were then precleared by incubation with protein A-agarose beads for 30 min and then the protein A-agarose removed by low speed centrifugation. To immunoprecipitate labeled proteins, 200 ng of anti-PDGFRα AF-307-NA or anti-EGFR LA1 were added to the clarified lysates to immunoprecipitate PDGFRα and EGFR, respectively. Lysates were incubated with antibodies for 2 h followed by protein A-agarose (50 µl) for an additional 2 h. The agarose beads were centrifuged at low speed, washed three times in 1% Triton X-100 in PBS, and eluted from the protein A-agarose by boiling for 5 min in 2×SDS loading buffer (100 mM Tris-Cl pH6.8, 20% glycerol, 4% SDS, 4% β-mercaptoethanol, 0.02% bromophenol blue) and separated by electrophoresis using 10% SDS-polyacrylamide gel.

### SDS-PAGE and Western blotting

ARPE-19 cell extracts were prepared by scraping cells into 1 ml PBS, centrifuging the cells at 800× g for 2 min and then lysing the cell pellet in 30 ul of protein extraction buffer (10 mM Tris-HCl pH 7.4, 5 mM EDTA, 50 mM NaCl, 1% triton X-100, 1 mM phenylmethylsulfonyl fluoride) and then centrifuged for 5 min at 13,000×g. The supernatant was removed and diluted in 2×SDS loading buffer and boiled for 5 min. A portion of the sample equal to 3×10^5^ cells was loaded into a single lane of a 10% SDS-polyacrylamide gel and separated by electrophoresis. Proteins were electrophoretically transferred to Immobilon membranes (Millipore) in a buffer containing 25 mM Tris, 192 mM glycine, and 20% methanol. Membranes were blocked in TBST (Tris-buffered saline with 0.1% tween-20 and 5% bovine serum albumin) for 30 min and then incubated TBST containing anti-PDGFRα 31614 or anti- β-actin (Sigma) diluted 1∶1000 and allowed to incubate at 4**°**C overnight. The blots were then washed three times with TBST and then incubated for 1 h in TBST with a 1∶1000 dilution of horseradish peroxidase-conjugated goat anti-mouse or mouse anti-rabbit IgG (Amersham). The blots were washed with TBST and proteins were visualized by incubating membranes in ECL chemiluminescent detection reagent (Pierce) for 60 sec and then exposing the membranes in an ImageQuant LAS 4000 detections system (General Electric).
